# Capturing T Lymphocytes’ Dynamic Interactions With Human Neural Cells Using Time-Lapse Microscopy

**DOI:** 10.3389/fimmu.2021.668483

**Published:** 2021-04-22

**Authors:** Florent Lemaître, Ana Carmena Moratalla, Negar Farzam-kia, Yves Carpentier Solorio, Olivier Tastet, Aurélie Cleret-Buhot, Jean Victor Guimond, Elie Haddad, Nathalie Arbour

**Affiliations:** ^1^ Department of Neurosciences, Faculty of Medicine, Université de Montréal, Montreal, QC, Canada; ^2^ Centre de Recherche du Centre Hospitalier de l’Université de Montréal (CRCHUM), Montreal, QC, Canada; ^3^ Centre Local de Services Communautaires des Faubourgs, Centre Intégré Universitaire en Santé et Services Sociaux du Centre-Sud-de-l’Ile-de-Montréal, Montréal, QC, Canada; ^4^ Centre de Recherche du Centre Hospitalier Universitaire Sainte-Justine (CHU Sainte-Justine), Department of Microbiology, Infectious Diseases, and Immunology and Department of Pediatrics, Faculty of Medicine, Université de Montréal, Montreal, QC, Canada

**Keywords:** astrocytes, neurons, inflammation, MHC class I, live imaging

## Abstract

To fully perform their functions, T lymphocytes migrate within organs’ parenchyma and interact with local cells. Infiltration of T lymphocytes within the central nervous system (CNS) is associated with numerous neurodegenerative disorders. Nevertheless, how these immune cells communicate and respond to neural cells remains unresolved. To investigate the behavior of T lymphocytes that reach the CNS, we have established an *in vitro* co-culture model and analyzed the spatiotemporal interactions between human activated CD8^+^ T lymphocytes and primary human astrocytes and neurons using time-lapse microscopy. By combining multiple variables extracted from individual CD8^+^ T cell tracking, we show that CD8^+^ T lymphocytes adopt a more motile and exploratory behavior upon interacting with astrocytes than with neurons. Pretreatment of astrocytes or neurons with IL-1β to mimic *in vivo* inflammation significantly increases CD8^+^ T lymphocyte motility. Using visual interpretation and analysis of numerical variables extracted from CD8^+^ T cell tracking, we identified four distinct CD8^+^ T lymphocyte behaviors: scanning, dancing, poking and round. IL-1β-pretreatment significantly increases the proportion of scanning CD8^+^ T lymphocytes, which are characterized by active exploration, and reduces the proportion of round CD8^+^ T lymphocytes, which are less active. Blocking MHC class I on astrocytes significantly diminishes the proportion of poking CD8^+^ T lymphocytes, which exhibit synapse-like interactions. Lastly, our co-culture time-lapse model is easily adaptable and sufficiently sensitive and powerful to characterize and quantify spatiotemporal interactions between human T lymphocytes and primary human cells in different conditions while preserving viability of fragile cells such as neurons and astrocytes.

## Introduction

Intravital and *in vitro* imaging studies have revealed that the spatiotemporal behavior of T lymphocytes is very dynamic. Activated T cells constantly patrol peripheral tissues and lymphoid organs looking for their cognate antigens. These leukocytes can respond to a plethora of soluble mediators as well as ligands expressed by local cells including antigen presenting cells (APCs) (1, 2). The interaction between the T cell receptor (TCR) on T lymphocytes and the major histocompatibility complex (MHC) on other cells represents a fundamental cellular interaction. Notably, T cells decelerate and arrest on isolated APCs harboring their cognate MHC-antigen complex (3). Two different types of *in vitro* T cell-APC interactions have been described: synapse and kinapse. A strong antigen recognition by the TCR triggers T cell migration arrest and long lasting interactions with MHC-expressing APC that is referred to as a synapse (4–6). Alternatively, interactions between the TCR and a low affinity MHC-peptide complex result in a more dynamic interaction referred to as a kinapse, with T lymphocytes exhibiting higher motility and transient contact with APC (5, 6). Beyond TCR-MHC/peptide complex interactions, multiple other factors shape the behavior of T cells and the combination of these factors can vary between organs and physiological conditions (ex. inflammation).

The central nervous system (CNS) carries multiple unique properties compared to other organs, including specific cell types such as astrocytes and neuron subsets (7). Infiltration of activated T cells into the CNS is observed under physiological conditions and has been associated with almost all neurodegenerative disorders (8). After crossing the blood brain barrier (BBB), infiltrating T cells migrate into the parenchyma and interact with neural cells such as astrocytes and neurons. T lymphocytes exert immune surveillance and can deploy both deleterious and beneficial functions depending on physiological and pathological contexts (9, 10). Notably, encephalitogenic T cells infiltrating the CNS during experimental autoimmune encephalomyelitis (EAE) exhibit an assortment of spatiotemporal behaviors within the same animal. Some T cells move very quickly (maximally 25µm/min) when migrating through the CNS parenchyma, while others show a very slow and short traveling distance and appear to spin around a fixed point (11). Moreover, encephalitogenic T cells can directly interact with all neural cell types, including astrocytes and neurons (9, 12). Multiple molecules have been implicated in the process of leukocytes crossing the BBB (13). However, the mechanisms and factors shaping the interactions between T lymphocytes that reach the CNS and local neural cells, as well as the consequences of these T cell-neural cell interactions, remain unclear.

Increasing evidence supports the notion that both the immune system and the CNS present species differences (14–16). To specifically tackle human T lymphocyte-neural cell interactions, we developed a co-culture assay to capture and characterize the interactions between activated human CD8^+^ T cells with primary human astrocytes and neurons. We took advantage of different fluorescent dyes not requiring transfection to label human astrocytes, neurons and CD8^+^ T lymphocytes. We characterized CD8^+^ T cell dynamics using time-lapse microscopy with high-quality spatiotemporal resolution. We used multiple variables extracted from CD8^+^ T cell tracking and showed that these cells exhibited different motility behaviors when in contact with astrocytes compared to neurons. To mimic *in vivo* inflammatory conditions, we treated astrocytes and neurons with the pro-inflammatory cytokine IL-1β prior to the addition of activated CD8^+^ T lymphocytes; we observed significant differences in CD8^+^ T cell motion induced by IL-1β-treatment. Finally, we show that blocking MHC class I on IL-1β-inflamed astrocytes impacts human CD8^+^ T cell dynamics and behavior. Our primary human co-culture assay can be easily adapted to other human primary cells and provides an excellent tool to investigate mechanisms of human cell-cell interaction.

## Material, Equipment and Methods

### Ethic Approvals

Written informed consent was obtained from all healthy donors for blood donation in accordance with the local ethical committees. Fetal (17-21 weeks) brain tissue was obtained after written informed consent (ethical committee of CHU Sainte-Justine, Montreal QC, Canada, CER#2126; University of Washington Birth Defects Research Laboratory Seattle, Washington, USA, STUDY00000380). These studies were approved by the Centre Hospitalier de l’Université de Montréal (CHUM) ethics boards (BH07.001, HD07.002).

### Isolation, Culture and Labeling of Human Neural Cells

Fetal brain tissues were processed to isolate human astrocytes and neurons using well established protocols (17–20). Briefly, tissue was washed with cold PBS, meninges and other debris were removed and then tissue was diced and finally digested with trypsin (0.25% v/v) (Life Technologies Thermo Fisher Scientific, Burlington ON, Canada, 15090-046), and DNAse (25 µg/ml; Worthington Biochemical, Lakewood New Jersey, LS002060) for 15 min at 37°C under agitation. Enzymatic digestion was stopped by adding fetal bovine serum (FBS), cells were filtered on a 132 μm nylon cell strainer and then washed in PBS. Dissociated mixed neural cells were plated in complete DMEM (ThermoFisher Scientific, 11995-065) containing 5% (v/v) FBS, penicillin 100 U/mL, streptomycin 100 µg/mL to which gentamycin sulfate 280 μg/ml (Wisent Bioproducts St-Bruno, QC Canada) and erythromycin 25μm/mL (Sigma-Aldrich, Oakville, ON Canada) were added on Poly-L-lysine coated flasks. After one week, medium was changed for gentamycin sulfate and erythromycin free complete DMEM. These mixed cultures became highly enriched in astrocytes with each subsequent passage, reaching 95% (according to GFAP staining) at passage 4 or 5 and being largely devoid of neurons and microglia as illustrated by qRT-PCR and immunocytochemistry (**Supplementary Figure 1**). One million astrocytes resuspended in 1 mL DMEM without red phenol (Wisent Bio product, # 319-050-CL) and FBS containing 10 µM of Cell Tracker Orange-CMRA dye (ThermoFisher Scientific, C34551) were incubated 15 min at 37°C in an Eppendorf tube. The dye was quenched by adding FBS for 3 min at room temperature and astrocytes were washed in DMEM. Orange-CMRA-labeled astrocytes were resuspended in imaging medium (red phenol-free DMEM containing 5% FBS, 2mM L-glutamine, penicillin 100U/mL, streptomycin 100µg/mL, and 20mM HEPES). Cells were seeded at 25,000 cells per chamber in 35mm imaging µ-dish with Quad polymer coverslip bottom and incubated 5-8 days prior to imaging.

For neuron isolation, a published protocol (21) was modified to deplete both MHC class I expressing cells and red blood cells. Dissociated neural cells obtained after digestion and filtration through a cell strainer were resuspended in MACS buffer (PBS-EDTA 2 mM, FBS 0.1%) and incubated 15 min at room temperature with anti-HLA-ABC-FITC antibody (BD Bioscience, Mississauga, ON, Canada, 555552, 5 µg/ml) and anti-CD235a-FITC (BD Bioscience, 559943, 50µg/ml). Cells were washed in MACS buffer and then incubated in MACS buffer containing anti-FITC microbeads (Miltenyi Biotec San Diego, CA USA, 130-097-050) for 15 min at 4°C. Cells were washed with MACS buffer and passed through a LS magnetic column (Milteniy Biotec, 130-042-401) according to the manufacturer’s instructions; the negative fraction was passed through a second LS column to increase purity. The final negative fraction containing neurons was collected and cultured in DMEM F12 without red phenol (ThermoFisher Scientific, 21041-025) containing 2% (v/v) MACS^®^ NeuroBrew^®^ (Miltenyi Biotec 130-093-566), penicillin 100 U/mL, streptomycin 100 µg/mL, 20mM HEPES. qRT-PCR performed on RNA isolated from purified neurons did not detect olig2 (oligodendrocyte lineage), GFAP (astrocytes), ITGAM (microglia) or CLDN5 (endothelial cells) even after 40 cycles (**Supplementary Figure 1A**). Isolated neurons expressed MAP2 and Tau as shown by immunocytochemistry (**Supplementary Figure 1C**). Neurons were plated onto poly-D-lysine (R & D Systems, Minneapolis, MN, USA, 3439-200-01) pre-coated 35mm imaging µ-dish with Quad polymer coverslip bottom (Ibidi, Fitchburg WI USA, 80416); 70,000 cells were plated per quadrant. About half of the medium was changed every 3-4 days. Neurons were kept in culture for a maximum of 12 days. One hour prior to imaging, neurons were labeled by adding 2 µM Taxol Janelia Fluor^®^ 646 (Tocris Bioscience, Oakville, ON Canada, 6266), a permeable dye that fluoresces only when bound to microtubules.

### qPCR

Fetal brain tissue or isolated cells (astrocytes or neurons) were put into TRIzol^®^ Reagent (Life technologies Thermo Fisher Scientic). Total RNA was extracted according to manufacturer’s instructions. RNA samples were transcribed into cDNA using Quantitect Reverse transcription kit (Qiagen, Mississauga, ON, Canada) as previously published (17, 18, 22). Relative gene expression levels were determined by quantitative real-time PCR (qPCR) using primers and TaqMan FAM–labeled probes GFAP (astrocyte marker), AQP4 (astrocyte marker), Olig2 (oligodendrocyte lineage marker), RBFox3 (neuronal marker) and ITGAM (microglial marker) and VIC–labeled probe for ribosomal 18S, the endogenous control, according to the manufacturer’s instructions (Applied Biosystems, Foster City, CA, USA). PCR was performed for 40 cycles.

### Immunocytochemistry

Astrocytes or neurons were fixed with 4% (w/v) paraformaldehyde, permeabilized with Triton (0.3%(v/v)) and blocked with goat and donkey sera. Antibodies targeting cell-specific markers: MAP2 (chicken anti-MAP2, ab5392 Abcam, Toronto ON, Canada), Tau (rabbit anti-human Tau, A002401-2 Agilent, Mississauga, ON, Canada), Iba1 (rabbit anti-Iba1, Fujifilm WAKO Chemicals, distributed by Cedarlane, Burlington, ON, Canada), or S100β (rabbit anti-S100β, ab52642 Abcam) were incubated overnight at 4°C in PBS containing 0.1% (v/v) Triton (PBS+T). After washes in PBS+T, Alexa Fluro^®^-488 or Alexa Fluro^®^-647 conjugated goat anti-rabbit or donkey anti-chicken (Jackson ImmunoResearch Labs, distributed by Cedarlane) were added for 1h at room temperature. Monoclonal mouse anti-GFAP conjugated to eFluor 615 (eBioscience, ThermoFisher Scientific) was added for 2 hours at room temperature. Finally, cells were incubated with 2-(4-Amidinophenyl)-6-indolecarbamidine dihydrochloride, 4′,6-Diamidino-2-phenylindole dihydrochloride (DAPI; Sigma Aldrich) and mounted in Gelvatol. Isotypes matched for concentration of primary Ab were used for all stainings. Slides were observed using a SP5 Leica confocal microscope and confocal images acquired sequentially in different channels using LASAF software.

### CD8^+^ T Cell Isolation, Activation and Labeling

Twelve healthy donors including 10 women and two men aged between 20 and 62 years old (median age 30.5 years old) were included in our study. Peripheral blood mononuclear cells (PBMC) were isolated from healthy donors’ blood using Ficoll density gradient as previously described (23–29). Briefly, blood samples were collected in EDTA-coated tubes (BD Biosciences). CD8^+^ T cells were isolated using CD8 Microbeads (Milteniy Biotech) according to the manufacturer’s instructions. CD8^+^ T cells were cultured in complete Iscove medium (ThermoFisher Scientific, 12440-053, FBS 10% (v/v), sodium pyruvate 1mM, L-glutamine 2mM, MEM nonessential amino acids 1%, β-mercapto-ethanol 1µM, penicillin 100U/mL, streptomycin 100µg/mL) and activated for 5 days on anti-CD3 (in house purified OKT3 clone, 17-50 μg/ml) pre-coated wells in the presence of anti-CD28 antibody (BD Biosciences 555725, 1µg/ml). After 5 days, activated CD8^+^ T cells were harvested and labeled with carboxyfluorescein succinimidyl ester (CFSE, 1.7mM, ThermoFisher, C34554) for 15 min at 37°C; CFSE was quenched with FBS for 3 min and cells washed in DMEM without red phenol prior to being resuspended in imaging medium described above.

### Co-Culture Assay

Astrocytes and neurons were either untreated or treated with recombinant human IL-1β (Life Technologies, PHC0815, 20ng/mL) for 24h. Before starting the co-culture, astrocytes were washed 2 times with imaging medium and 100,000 CFSE labeled CD8^+^ T cells were carefully added to the culture (T cell: astrocyte ratio, 4:1). As neurons are sensitive to a complete change of media, 100,000 CFSE labeled CD8^+^ T cells (T cell:neuron ratio 10:7) were added directly into the culture without media changes. The co-culture was imaged after 10 to 15 min to allow CD8^+^ T cells to fall by gravity onto the CNS cell monolayers. For MHC class I blocking assays, anti HLA-ABC monoclonal antibody (W6/32) (Thermo scientific, 16-9983-85) or IgG2a kappa control isotype (Thermo scientific, 14-4724-85) were added to washed IL-1β-treated astrocytes at 50μg/mL for 30 min before adding CFSE labeled CD8 T cells for co-culture.

### Imaging Set Up

Co-cultures were imaged with a Zeiss AxioObserver Z1 Yokogawa CSU-X1 Spinning disk confocal microscope equipped with a motorized stage and Piezo objectives and an Evolve EMCCD (512x512, 16bit) monochrome camera (Photometrics). Co-cultures in 35mm imaging µ-dish Quad polymer were placed in a 37°C, 5% CO_2_ top chamber (Tokai Hit). The Fluar 40X/1.3 Oil M27 objective (free WD 0.16mm; Zeiss) was also maintained at 37°C using a lens heater. To eliminate Z drift during the experiment, the Definite Focus module was used. Samples were illuminated with 488nm (20mW) and 561nm (50mW) solid state lasers for astrocytes and 488nm and 633nm solid state lasers for neurons. To prevent cell phototoxicity, laser power was set as low as possible (<10%) and the total power was filtered through a beam-splitter dichroic mirror to emit only 20% of the light source at the objective.

### Image Analysis

*Post-acquisition processing:* 3D blind deconvolution processing using AutoQuant X3 software (Media Cybernetics) was performed using the dyes’ specificities, lasers and physical properties of the microscope. This method adapts itself to the real point spreading function (PSF) of the microscope system due to specimen and instrument variations. Deconvolution settings use 10 iterations and medium noise. AutoQuant’s image restoration improves both the image resolution and its contrast, leading to enhanced visualization and post-acquisition analysis.

*CD8 T lymphocyte tracking:* Deconvoluted images were exported to Imaris software (V9.6 Bitplane, Oxford Instruments group) to analyze CD8^+^ T cell behavior co-cultured with astrocytes or neurons. The loss of fluorescent signal due to repetitive laser exposure (laser 488 and 561 or 633 nm, 21 Z-slices, 1 image/min over 2h) was compensated by the fluorescence normalization over time using the MATLAB plugin XTM Normalize Time Points of Imaris. CD8^+^ T cell movements were tracked using Imaris spot object algorithm based on the source channel 488 and to create tracks for each cell. Tracks exhibiting less than 300s (5 time points) duration in the imaged field were excluded. All cell tracks were manually corrected using the manual editor when misdetection or non-detection were observed. Despite selecting a size threshold of 6-7 uM and embedded quality threshold for moving objects, smaller debris or dead cells were captured by the software. Sometimes, multiple tracks were attributed to one single living cell and had to be fused to fully capture the movement of that cell. Within the 2-hour acquisition, tracks of cells presenting signs of cell death based on their morphology were stopped at first sign of blebbing; these dying cells represented 3-6% of the captured tracks. Overall, excluded or corrected tracks represented 30-50% of the by-default captured tracks. As neurons could not be washed prior to CD8 T cell addition and co-culture, the Taxol Janelia Fluor^®^ 646 also stained T cells’ polymerized microtubules. A third channel (white) corresponding to the colocalization of CFSE and Taxol Janelia Fluor^®^ 646 signal (CD8^+^ T cells polymerized microtubules) was artificially added to all images using the colocalization function of Imaris.

Nine numerical variables were collected to characterize CD8 T cell motion over time (**Table 1**, #1-9). Four CD8^+^ T lymphocyte behaviors (scanning, dancing, poking and round) were visually identified based on cellular shapes and general movements. For each CD8^+^ T cell track, one of the four behaviors was attributed to each time point over the entire track, identifying the major behavior characterizing each cell. The global behavior attributed to the entire cell track corresponded to the behavior observed for the longest period of time. A behavior change value representing the number of time one particular cell changed its behavior over time was attributed to each CD8^+^ T cell track. The nine spatiotemporal numerical variables and the behavior change value were normalized to the mean and standard deviation of each variable using the statistical software R before being associated to the general behavior characterizing each CD8^+^ T cell track. UMAP clustering using the R *umap* package was performed to determine the repartition of the different behaviors using their spatiotemporal characteristics. Heat maps of Pearson’s pairwise correlations were generated using R and *pheatmap* package.

**Table 1:** Parameters collected from individual CD8^+^ T lymphocytes by time-lapse microscopy.

	Parameter (unit)	Description
1	Track duration (min)	Duration between the first and the last time point within the tracks
2	Track length (μm)	Total length of displacement within the track
3	Displacement (μm)	Distance between the first and the last cell positions
4	Track straightness	Cell’s displacement divided by the track length
5	Track speed max (μm/min)	Maximum value of the cell’s speed over its track
6	Track speed mean (μm/min)	Track length divided by the time between the first and the last object within the track
7	Track speed min (μm/min)	Minimum value of the cell’s speed on the track
8	Speed variation	Ratio of the standard deviation value of the cell’s speed on the track to the track speed mean value
9	Arrest coefficient (%)	Proportion of time the cell’s instantaneous speed is less than 2 μm/min over its entire track and represents the proportion of time a cell is considered as not moving
10	Behavior changes	Number of behavior changes observed over the entire track of each cell.

### Statistical Analysis

Data analysis was performed using the statistical software R. When data passed the Shapiro-Wilk normality test, t-test comparison was used to compare conditions between two groups. When data did not pass the normality test, Wilcoxon test comparison test was used. For the behavior proportions, data analysis was performed using Prism 9 software (GraphPad, La Jolla, CA, USA). When data passed the Shapiro-Wilk or the Kolmogorov-Smirnov normality test, ANOVA followed by Dunnett’s multiple comparison test was used. Values were considered statistically significant when probability (P) values were equal or below 0.05 (*), 0.01 (**), 0.001 (***) or 0.0001 (****).

## Results

### Co-Cultures of Human T Cells and Primary Human Astrocytes and Neurons Can Be Efficiently Imaged

**Figure 1:**
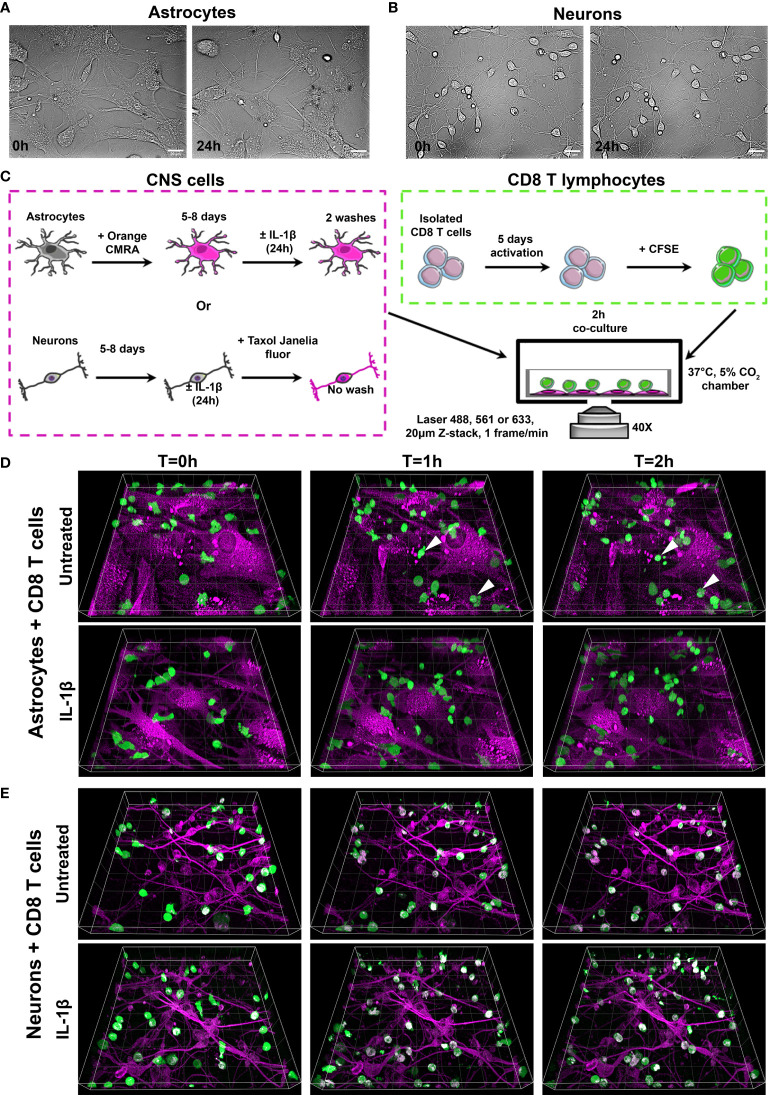
Efficient time-lapse imaging of human CD8^+^ T cells co-cultured with primary human astrocytes and neurons. **(A, B)** Representative bright field images of primary human astrocytes (**A**, **SI movie 1**) and neurons (**B**, **SI movie 2**) at 0h and 24h in the microscope incubation chamber (37°C, 5% CO_2_). **(C)** Experimental procedure for CD8^+^ T cell-astrocyte and CD8^+^ T cell-neuron co-cultures. Human astrocytes were labeled with Orange CMRA dye and cultured 5 to 8 days before being treated or not with IL-1β for 24h. Unlabeled human neurons were cultured 5 to 8 days before being treated or not with IL-1β for 24h. In parallel, isolated human CD8^+^ T cells were activated with αCD3/αCD28 antibodies for 5 days. Activated CD8^+^ T cells were labeled with CFSE prior to being added to washed human astrocytes. For CD8 T cell-neuron co-cultures, neurons were labeled with Taxol Janelia Fluor^®^ 646 for 1h, after which CFSE labeled CD8^+^ T cells were added. Cells were imaged in the microscope incubation chamber every minute for 2h. **(D, E)** Three dimensional time lapse view at T=0, T=1 and T=2h of activated CD8^+^ T cells (green) co-cultured on untreated or IL-1β-treated astrocytes (**D**, **SI movies 3** and **4**) or neurons (**E**, **SI movies 5** and **6**) (magenta). **(D)** White arrows point to rare CD8^+^ T cells undergoing cell death. **(E)** A third channel (white) corresponding to CFSE and Taxol Janelia Fluor^®^ 646 colocalization in CD8^+^ T cells (polymerized microtubule) was artificially added post acquisition. Gridlines=20 µm.

Our ultimate goal is to investigate how local CNS inflammation impacts the complex interactions between human neural cells and incoming human T cells. To examine the multifaceted exchanges between human CD8^+^ T cells and astrocytes and neurons, we developed a co-culture assay combined with time-lapse spinning-disc microscopy to efficiently capture spatiotemporal interactions. We used highly enriched human primary cultures of astrocytes and neurons (**Supplementary Figure 1**). To ensure that cell physiology and viability of primary human neural cells were not affected by our imaging conditions, astrocytes and neurons were placed in 37°C, 5% CO_2_ chamber and imaged under bright field illumination every hour over 24h (**Figure 1A** and **SI Movies 1** **,** **2**). We did not observe abnormal changes in astrocyte (**Figure 1A** and **SI Movie 1**) or in neuron (**Figure 1B** and **SI Movie 2**) shape and viability. To develop an assay easily adapted to multiple cell donors and to avoid potential cellular stress triggered by transfection or viral vectors, we labeled cells with permeable and non-toxic fluorescent dyes to easily distinguish neural cells and lymphocytes while performing time-lapse acquisition to capture T cell behavior.

Astrocytes were labeled with Orange CMRA dye that mostly stained cytoplasmic proteins and allowed excellent visualization of astrocytes (**Figure 1C**). After 5 to 8 days, labeled astrocytes were treated or not for 24h with IL-1β, an inflammatory mediator shown to be upregulated in the context of multiple conditions such as brain injury, neurodegenerative diseases and infections (30–32). In parallel, isolated CD8^+^ T cells were activated for 5 days *in vitro* using the well-established anti-CD3/anti-CD28 antibody paradigm to mimic the peripheral activation of CD8^+^ T cells prior to their migration to the CNS. As T cells rapidly divide upon activation and consequently would decrease any initial dye content, these cells were labeled after their activation, just prior to their addition to neural cultures (**Figure 1C**). Untreated or IL-1β-treated astrocytes were washed to remove the inflammatory cytokine prior to the addition of CFSE-labeled activated T cells.

A similar protocol was optimized for human neurons. As neuronal viability and adherence were affected by removing medium, we used Taxol Janelia Fluor^®^ 646, a dye that fluoresces only when bound to polymerized microtubules, to avoid extra washing steps (**Figure 1C**). The dye was added to untreated or IL-1β treated neurons one hour prior to adding CFSE-labeled CD8^+^ T cells (**Figure 1C**). Representative images showing 3 time points (0, 1h and 2 h) acquired from co-cultures of untreated or IL-1β-treated astrocytes or neurons with CFSE-labeled CD8^+^ T cells show that T lymphocytes were easily distinguished from astrocytes or neurons (**Figures 1D, E**). Moreover, CD8^+^ T cell movements in astrocyte (**SI Movies 3A, B**) or neuron (**SI Movies 4A, B**) co-cultures were captured with adequate spatial and temporal resolution. We did not observe apparent photo-toxicity for astrocytes, neurons and CD8^+^ T cells during the 2h time lapse. Astrocyte or neuron death was not observed during imaging. We detected rare CD8^+^ T cells exhibiting cell blebbing (**Figure 1D** white arrow) suggesting that they were undergoing cell death. However, we concluded that photo-toxicity was minimal as cell death was sparse and not present in all time lapses. Dead cells were removed from analysis.

### Inflammation Shapes the Spatiotemporal Characteristics of Human T Cells on Primary Human Neural Cells

**Figure 2:**
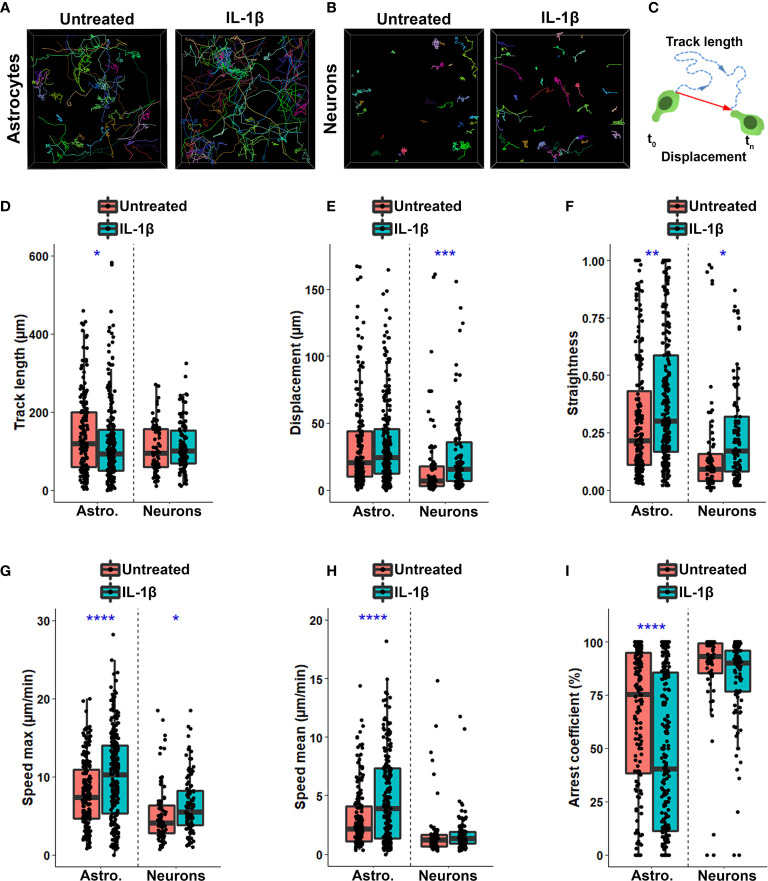
Inflammation shapes spatiotemporal characteristics of human CD8^+^ T cells interacting with primary human neural cells. **(A, B)** Representative cell tracks of CD8^+^ T cells cultured on resting or IL-1β-treated astrocytes **(A)** or neurons **(B)**. Each individual track is associated with one color. **(C)** Schematic representation of track length (blue dashed line) corresponding to total length of displacement and displacement length (red vector) being the distance between the first and the last cell positions. **(D-I)** Box plot presenting spatiotemporal variables measured for each individual CD8^+^ T lymphocyte tracked on untreated (red) or IL1β-treated (blue) astrocyte or neurons over 2h. Line represents median in each box. Each dot represents one cell. **(D)** Track length (μm); **(E)** Displacement length (μm); **(F)** Straightness of CD8^+^ T cell migration; **(G)** Maximal speed (μm/min); **(H)** Speed mean of CD8^+^ T cell measured along their track (μm/min); **(I)** Arrest coefficient (%). Data for astrocyte-CD8^+^ T cell co-cultures are pooled from 5 distinct experiments; for each experiment a unique donor of astrocytes and a unique donor of CD8^+^ T cells were used. For each astrocyte-CD8 T cell co-culture condition between 18 and 65 CD8^+^ T cell tracks were collected for a total of 404 tracked CD8^+^ T cells. Data for neuron-T cell co-cultures are pooled from 4 distinct experiments; for each experiment a unique donor of neurons and a unique donor of CD8^+^ T cells were used. For each neuron-CD8^+^ T cell co-culture condition, 11 to 40 CD8^+^ T cell tracks were collected for a total of 177 tracked CD8^+^ T cells. Untreated vs. IL1β-treated conditions *P < 0.05, **P < 0.01, ***P < 0.001, ****P < 0.0001.

To examine how astrocytes and neurons and their exposure to inflammatory mediators such as IL-1β shape the behavior of human CD8^+^ T cells, we tracked individual CD8^+^ T cells during a 2h time lapse to capture their spatiotemporal dynamics (**Figures 2A, B**). Astrocytes are relatively large cells and closely interact with each other creating a dense cell monolayer with very few uncovered surface. CD8^+^ T cells added to these glial cells were thus usually in contact with one or more astrocytes. Neurons are smaller cells and despite the important network formed by their processes, they do not cover the entire surface of the dish at the optimal density for their survival. Consequently, the proportion of CD8^+^ T cells not actively interacting with neural cells during the recorded time lapse was greater in neuron co-cultures than astrocyte counterparts. Hence, CD8^+^ T cells that were in direct contact with neurons for less than 10% of the time lapse tracking were excluded from the analysis. As T lymphocytes randomly entered and exited the imaged field, cell tracks with a track duration under 300s were ignored. We excluded small debris and T cells exhibiting abnormal shapes or signs of fragmentation. The behavior of activated CD8^+^ T cells from one donor on untreated vs. IL-1β treated astrocytes or neurons were recorded on the same neural cell donor, on the same day with similar microscope settings. Finally, several independent experiments using different human donors of astrocytes, neurons and T cells were performed and spatiotemporal characteristics from individual CD8^+^ T cells assessed in these multiple experiments are shown (**Figure 2**).

We observed that most CD8^+^ T cells in contact with untreated astrocytes exhibited a limited exploration of their neighboring environment as illustrated by color coded individual tracks (**Figure 2A**). However, when astrocytes had been pre-treated with IL-1β, the proportion of CD8^+^ T cells widely exploring their neighboring environment was increased (**Figure 2A**). In contrast, CD8^+^ T cells showed a reduced exploring behavior upon contact with human neurons compared to astrocytes (**Figures 2A, B**); they were more confined to small areas with long and stable periods of interactions with neurons. IL-1β pre-treatment of neurons also increased CD8 T cell exploratory behavior but less extensively than for IL-1β-treated astrocytes (**Figure 2B**).

Multiple spatiotemporal measures (**Table 1**, variables 1-9) of CD8^+^ T lymphocyte motion were altered in IL-1β-treated conditions compared to untreated counterparts. The track length of CD8^+^ T cells in contact with IL-1β-treated astrocytes was significantly reduced compared with lymphocytes on untreated glial cells (**Figures 2C, D**) despite similar global displacement over time in both conditions (**Figures 2C, E**). Reduction in track length but not displacement led to a significant increase in the straightness of the traveling path of T cells, which means that CD8^+^ T cells had less directional changes when added to IL-1β-treated astrocytes compared to untreated cells (**Figure 2F**). In contrast, CD8^+^ T cells in contact with IL-1β-treated neurons showed a similar track length to counterparts on untreated neurons but a significantly greater displacement length, also resulting in an increase of the straightness of the traveling path (**Figures 2D–F**).

We also evaluated whether IL-1β treatment impacts the speed of T cells in contact with astrocytes and neurons. For both astrocyte-CD8 and neuron-CD8 co-cultures, the maximum speed achieved by individual CD8^+^ T cells was significantly increased when neural cells had been pre-treated with IL-1β compared to untreated counterparts (**Figure 2G**). Moreover, CD8^+^ T cells showed a greater average speed when co-cultured on IL-1β-treated astrocytes versus untreated counterparts (**Figure 2H**). However, the average speed of CD8^+^ T cells was similar in both untreated and IL-1β-treated neuron co-cultures (**Figure 2H**). Finally, we observed a significant decrease in the arrest coefficient (**Table 1**) for CD8^+^ T cells in contact with IL-1β-treated astrocytes compared to untreated glial cells (**Figure 2I**), reflecting a global increased of cell motility. CD8^+^ T cells had similar arrest coefficients on resting and IL-1β-treated neurons (**Figure 2I**). Our observations suggest that inflammation, which may be mediated by IL-1β treatment, alters the spatiotemporal behavior of activated CD8^+^ T cells interacting with either astrocytes or neurons. Finally, our time-lapse assay was sufficiently refined to capture these differences.

### Human T Cells Exhibit Distinct Movement Profiles Upon Co-Culture With Astrocytes

**Figure 3:**
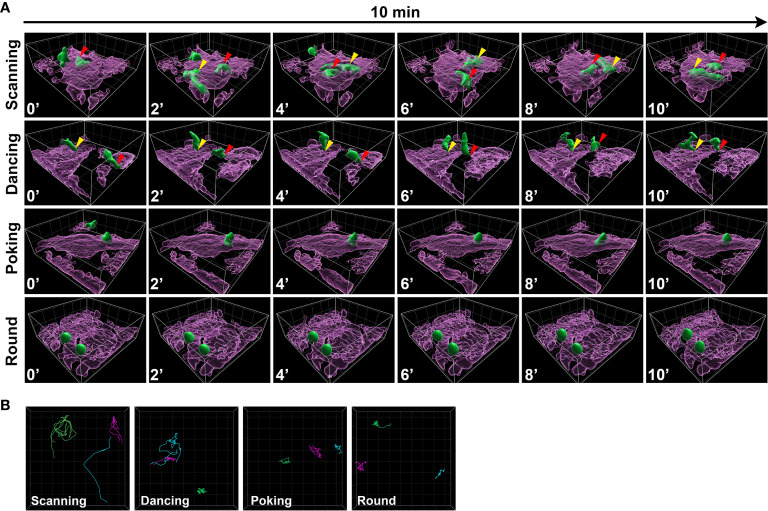
CD8^+^ T cells co-cultured on astrocytes exhibit four different movement behaviors. **(A)** Three dimensional time lapse view captured over 10 min of surfaced astrocytes (magenta) and CD8^+^ T cells (green), showing a representative example of each CD8^+^ T lymphocyte behavior: scanning (**SI movie 7**), dancing (**SI movie 8**), pocking (**SI movie 9**) and round (**SI movie 10**). The relative time is indicated in minutes. Gridlines= 10 µm. The same moving cell is identified with a specific colored arrow (red, yellow) in different frames. **(B)** Representation of 3 CD8^+^ T cell tracks for scanning, dancing, poking and round behavior; each cell track is identified by a different color.

Upon their interaction with astrocytes, we observed that T cells exhibited heterogeneous spatiotemporal behaviors. Values of T cell dynamic characteristics (e.g. displacement, straightness, and coefficient of arrest) were relatively heterogeneous (**Figures 2D–I**) suggesting that T cells explore their environment *via* different behaviors. We visually identified four distinct subsets of CD8^+^ T cells based on their shape and global spatial exploration and named them scanning, dancing, poking and round. *Scanning* CD8^+^ T cells were characterized by a flattened shape and elongated uropod, as represented by the 3D surface reconstruction (**Figure 3A** and **SI Movie 5**). They also presented hallmarks of fast amoeboid migration, previously reported for T cells in kinapse-like transient interaction (4, 33, 34). Scanning cells quickly changed direction, actively explored their neighboring environment, crawled on top of astrocytes and were able to migrate underneath astrocytes (**Figures 3A, B** and **SI Movie 5**). *Dancing* CD8^+^ T cells exhibited an elongated shape with one uropod attached to the astrocyte membrane on a very focal point. The cell body oscillated around this contact point (yellow arrow). Dancing T cells were able to detach from their first contact point to re-localize and re-attach (red arrow) to a different anchor (**Figures 3A, B** and **SI Movie 6**). *Poking* CD8^+^ T cells had diverse cell morphologies and actively explored a limited local environment by vigorously pushing pseudopods through astrocyte membranes (**Figures 3A, B** and **SI Movie 7**). These CD8^+^ T cells actively extended membrane protrusions underneath astrocytes and even slowly glided under the astrocyte monolayer while still actively moving their protrusion in different directions. *Round* CD8^+^ T cells had globally round shape with no apparent membrane protrusion but engaged in stable interactions with astrocytes over time (**Figures 3A, B** and **SI Movie 8**). Both poking and round CD8^+^ T cell movement profiles reflect more stable interactions and less exploration of their neighboring environment, which can be related to a synapse-like interaction (4, 33, 34). Importantly, we noticed that some cells switched from one behavior to another during the 2h live imaging, suggesting that CD8^+^ T cells can adapt their behavior over time while interacting with astrocytes.

**Figure 4:**
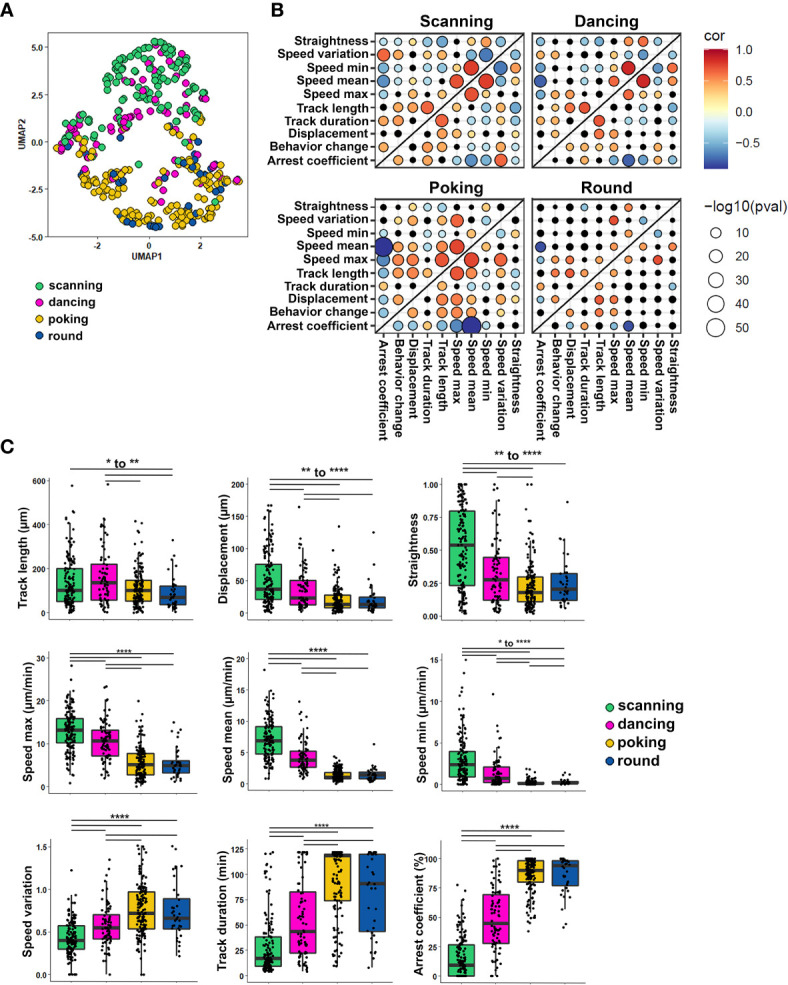
Each CD8^+^ T cell motion behavior exhibits a unique combination of spatiotemporal properties. The spatiotemporal characteristics of 404 tracked CD8^+^ T cells co-cultured with human resting and IL-1β-treated astrocytes from 5 independent experiments were analyzed and compared between the four CD8^+^ T cell motion behaviors: scanning, dancing, poking and round. **(A)** UMAP visualization of CD8^+^ T cell behaviors using 10 spatiotemporal track variables measured for each cell. CD8^+^ T lymphocyte behavior was assigned to respective cell tracks according to visual criteria and plotted on the UMAP. **(B)** Heat map of pairwise correlations for each behavior and parameter. Dot size indicates –log10 of the p value and correlation coefficient values are color coded as indicated. **(C)** Box plot presenting individual spatiotemporal variables for each behavior: track length (μm), displacement (µm), straightness, speed mean (µm/min), speed variation and arrest coefficient (%) are presented. Line indicates median. Comparison between each behavior *P < 0.05, **P < 0.01, ***P < 0.001, ****P < 0.0001.

To investigate whether collected quantitative variables of individual cell tracks could discriminate the four CD8^+^ T cell behaviors, we labeled each T cell track (404 CD8^+^ T cells) with the behavior observed for the longest proportion of time and used the numerical variables associated with each track to perform a dimension reduction. In total, nine numerical parameters describing CD8^+^ T cell motion (**Table 1**, #1-9) were obtained using Imaris software analysis. As cells were labeled according to their predominant behavior, we also included the number of times cells changed their behavior as a tenth numerical feature (**Table 1**, #10). Uniform Manifold Approximation and Projection (UMAP) was used to visualize how these ten quantitative features could segregate CD8^+^ T cell behaviors regardless of astrocyte treatment. This approach showed that scanning CD8^+^ T cells (green dots) clearly separated from poking (yellow dots) and round (blue dots) CD8^+^ T cells (**Figure 4A**). Round and poking CD8^+^ T cells showed similar distribution and could not be easily distinguished based on the UMAP. Finally, dancing (pink dots) CD8^+^ T cells showed a more spread distribution and did not separate as clearly based on combined spatiotemporal values (**Figure 4A**). This visualization of all collected spatiotemporal features suggests that numerical characteristics extracted from observed tracks mostly segregate kinapse- (scanning) and synapse-like (poking and round cells) behaviors.

We also investigated whether specific correlations between the collected parameters vary between cell behaviors (**Figure 4B**). The heat maps of correlation coefficients show strong positive and negative correlations especially for scanning, poking and dancing CD8^+^ T cell behaviors (**Figure 4B**); in contrast, fewer correlations reached significance for round cells. As expected, minimal and maximal speed positively correlated with mean speed for all behaviors (**Figure 4B**). A strong positive correlation between speed variation and arrest coefficient and conversely a negative correlation between speed variation and track straightness and mean speed were observed for scanning and dancing CD8^+^ T cells. These parameters showed no relationship for poking and round cells. In contrast, a negative correlation was observed between displacement length and arrest coefficient for poking and round CD8^+^ T cells whereas these parameters had no significant relationship for scanning and dancing cells. These comparisons support that CD8 T cell behaviors split into two major groups; the first group includes scanning and dancing CD8^+^ T cells which exhibited kinapse-like behavior. The poking and round CD8 T cells form the second group and showed synapse-like behavior. Nevertheless, each behavior exhibited unique characteristics. For example, a positive correlation between track length and speed mean was only observed for poking CD8^+^ T cells. Dancing CD8^+^ T cells were the only cell subset that did not exhibit a positive correlation between track length and maximal speed. Scanning CD8^+^ T cells did not show the positive correlation between speed variation and maximal speed observed in all other subsets. The combined UMAP and heat maps of correlation coefficients of numerical objective measures support that each of the four CD8^+^ T cell motion behaviors exhibit a unique combination of properties.

The analysis of individual features across annotated behaviors showed specific patterns. Scanning CD8^+^ T cells presented the highest velocity values (speed max, speed mean, speed min) (**Figure 4C**). Their displacement was longer and straighter than all other CD8^+^ T cells’ behaviors. They also presented lower speed variation which correlated with a smaller arrest coefficient compared to dancing, poking and round CD8^+^ T cells. These characteristics support the idea that scanning CD8^+^ T cells interact with astrocytes in a kinapse-like manner, highly exploring their environment. In contrast, poking and round CD8^+^ T cells were very similar and could not be distinguished based on individual measured parameters. These two populations globally presented low velocity, and their displacement was smaller and less straight than scanning and dancing CD8^+^ T cells (**Figure 4C**). They also presented more speed variation, which was associated with a higher arrest coefficient (**Figure 4C**). These results suggest that poking and round CD8^+^ T cells presented synapse-like characteristics upon interacting with astrocytes. Dancing CD8^+^ T cells presented numerical spatiotemporal values lying between those of scanning and poking/round CD8^+^ T cells (**Figure 4C**). They exhibited a lower motility profile than scanning but had higher motility than poking and round T cells. Nevertheless, our UMAP and heat maps of correlation coefficients strongly suggest that dancing CD8^+^ T cells present greater similarity to scanning CD8^+^ T cells than to poking/round counterparts. Overall, our astrocyte-CD8 T lymphocyte co-culture assay could distinguish four types of CD8^+^ T lymphocyte behaviors that harbor either kinapse or synapse-like characteristics.

Finally, CD8^+^ T cells exhibited more limited exploring behaviors upon their contact with human neurons compared to astrocytes. Visual distinction of the four CD8^+^ T cell behaviors was less clear in neuron co-cultures compared with astrocyte co-cultures. The UMAP representation demonstrates that CD8^+^ T lymphocyte behaviors had less specific spatiotemporal quantitative features when interacting with neurons (**SI Figure S1-A**).

### Inflammation Alters the Spatiotemporal Behavior of Human T Cells Co-Cultured With Astrocytes

**Figure 5:**
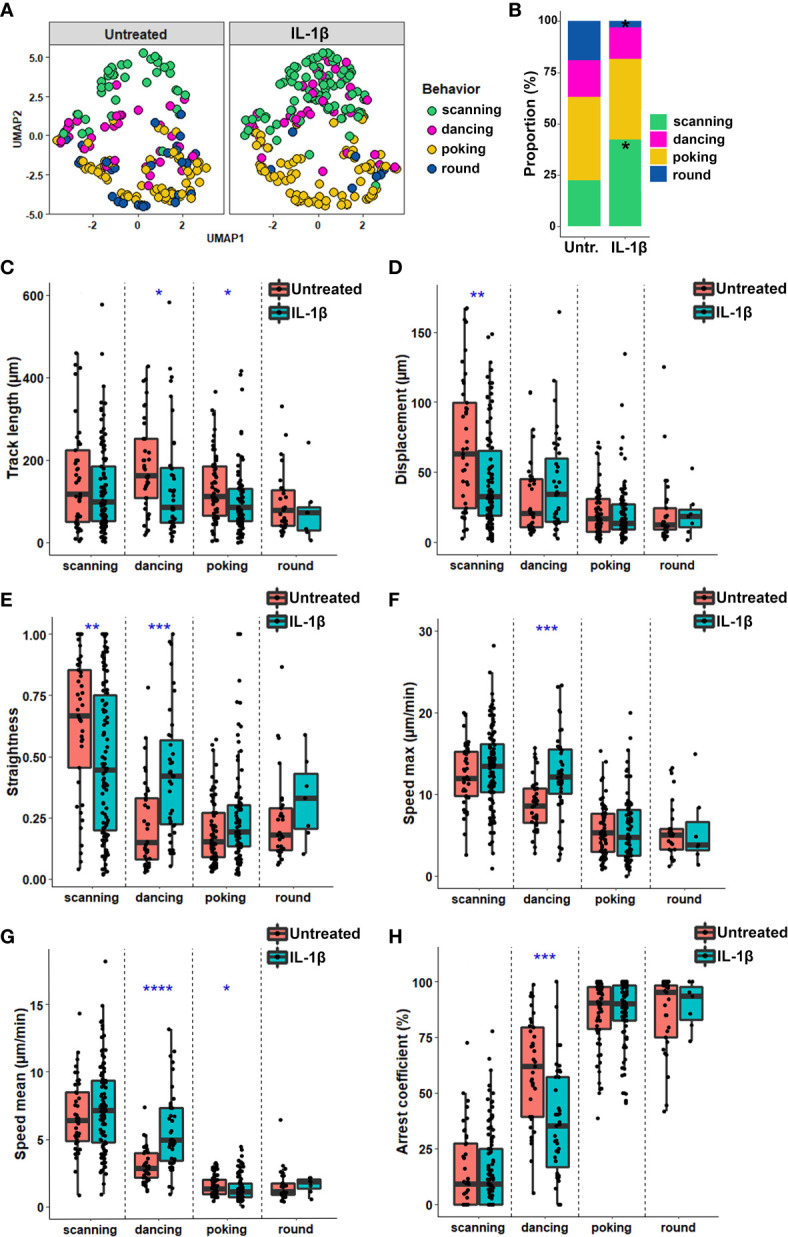
Inflammation alters the spatiotemporal behavior of human CD8^+^ T cells co-cultured with astrocytes. **(A)** UMAP analysis of 10 spatiotemporal variables measured for CD8^+^ T cells co-cultured on untreated or IL-1β-treated astrocytes according to four behaviors. **(B)** Proportion of CD8^+^ T cells exhibiting the scanning, dancing, poking and round behaviors when co-cultured with untreated or IL1β-treated astrocytes. **(C–H)** Box plot representing the spatiotemporal variables for each individual CD8 T cell track according to their behavior when co-cultured with untreated or IL1β-treated astrocytes. Line indicates median. **(C)** Track length (μm); **(D)** Displacement length (μm); **(E)** Straightness of CD8^+^ T cell migration; **(F)** Maximal speed (μm/min); **(G)** Speed mean of CD8^+^ T cell (μm/min); **(H)** Arrest coefficient (%). Statistical comparison untreated vs. IL-1β: *P < 0.05, **P < 0.01, ***P < 0.001, ****P < 0.0001. Data for astrocyte-CD8^+^ T cell co-cultures are pooled from 5 distinct experiments; for each experiment a unique donor of astrocytes and a unique donor of CD8^+^ T cells were used. For each astrocyte-CD8^+^ T cell co-culture condition between 18 and 65 CD8^+^ T cell tracks were collected for a total of 404 tracked CD8^+^ T cells.

To determine whether inflammation of glial cells impacts specific spatiotemporal behaviors of CD8^+^ T cells, we applied the same analysis (**Figure 4**) to T cells co-cultured on untreated vs. IL-1β-treated astrocytes. We observed a similar distribution of the scanning, poking and round behavioral groups of CD8^+^ T cells with clear separation of kinapse-like and synpase-like behaviors (**Figure 5A**) in both co-culture conditions. However, the proportion of CD8^+^ T cells adopting the scanning behavior (green dots) significantly increased (23.4% to 44.5%) while the percentage of T cells showing the round (blue dot) behavior decreased (16.6% to 3.1%) in IL-1β-treated astrocyte co-cultures compared to untreated counterparts (**Figure 5B**). Comparison of CD8^+^ T cells in contact with IL-1β-treated vs. untreated neurons did not reveal significant differences in behavior proportions (**Figures S1 B, C**). The majority of CD8^+^ T cells in co-culture with neurons exhibited poking or round behaviors and very few cells showed the scanning behavior regardless of neuronal treatment (**Figures S1 B, C**). Our results suggest that astrocytes have a greater impact on CD8^+^ T cell motility than neurons, and that how astrocytes respond to IL-1β stimulation in turn modifies CD8^+^ T cell behavior.

To investigate whether specific parameters of CD8^+^ T lymphocyte behavior subsets were affected by inflamed astrocytes, we compared the cell track characteristics of the four different behaviors in both untreated and IL-1β-treated astrocytes. Most characteristics were similar for poking and round cells in both conditions (**Figures 5C–H**). In contrast, scanning T cells on IL-1β-treated astrocytes exhibited shorter displacement (**Figure 5D**) and reduced straightness (**Figure 5E**) compared with those on untreated astrocytes. These observations suggest that although the track length of scanning CD8^+^ T cells was similar, these cells more actively explored a smaller immediate environment when in contact with inflamed versus untreated astrocytes. The characteristics of dancing CD8^+^ T cells were the most strongly affected by contact with IL-1β-treated astrocytes. These cells presented significantly reduced track length and arrest coefficient and increased displacement, straightness, and speed (max and mean) when co-cultured with IL-1β-treated versus untreated astrocytes (**Figures 5C–H**). Overall, our findings show that CD8^+^ T cells adapt their behavior when interacting with IL-1β-inflamed astrocytes by increasing the scanning and decreasing the round cell proportions. IL-1β-induced inflammation also increased dancing CD8^+^ T cell dynamics, promoting a more exploratory motion.

### MHC Class I Expression by Astrocytes Favors Specific CD8^+^ T Cell Spatiotemporal Behaviors

**Figure 6:**
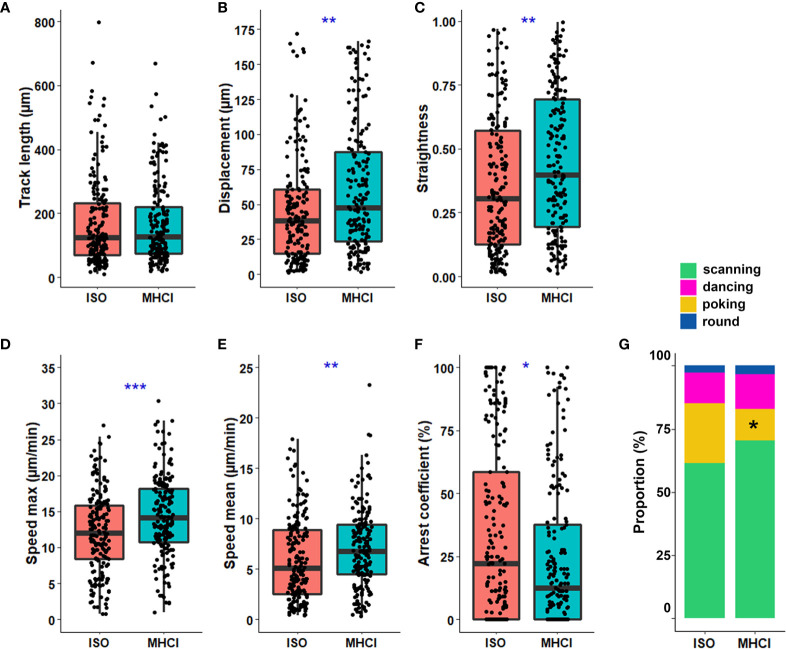
MHC class I expression by astrocytes favors poking behavior by CD8^+^ T cells. IL-1β-inflamed astrocytes were pre-treated with anti-MHC class I blocking antibody (MHCI) or isotype (ISO) for 30 min before the addition of activated CD8^+^ T cells. **A–F**) Boxplot presenting the track length (μm) **(A)**, displacement (μm) **(B)**, straightness **(C)**, maximal speed (μm/min) **(D)**, mean speed (μm/min) **(E)**, and the arrest coefficient (%) **(F)** for each tracked CD8^+^ T cell. **(G)** Proportion of CD8^+^ T cells exhibiting the scanning, dancing, poking and round behaviors in anti-MHC class I or isotype treated astrocyte co-cultures. Anti-MHC class I vs. isotype *P < 0.05, **P < 0.01, ***P < 0.001. Data for astrocyte-CD8^+^ T cell co-cultures are pooled from 3 distinct experiments; for each experiment a unique donor of astrocytes and a unique donor of CD8^+^ T cells were used. For each astrocyte-CD8^+^ T cell co-culture condition between 39 and 76 CD8^+^ T cell tracks were collected for a total of 363 tracked CD8^+^ T cells.

The interaction between the TCR on T cells and the MHC class I complex on APC shapes the dynamics of CD8^+^ T cells patrolling lymphoid organs and peripheral tissues (1). To investigate how the TCR –MHC class I interaction impacts CD8^+^ T cell spatiotemporal behaviors, we blocked MHC class I on IL-1β-treated astrocytes [which express elevated levels of MHC class I (20)] and performed our extensive CD8^+^ T lymphocyte tracking analysis. We observed a significant increase in CD8^+^ T cell displacement and travel path straightness when MHC class I was blocked compared to the isotype condition (**Figures 6B, C**). Moreover, CD8^+^ T cells exhibited significantly elevated speeds (maximal and mean; **Figures 6D, E**) which correlated with a reduced arrest coefficient (**Figure 6F**) when the TCR-MHC class I interaction was prevented compared with control. To determine whether the TCR-MHC class I interaction plays a role in specific spatiotemporal behaviors we described in Fig 3-4, we characterized the proportion and parameters for each behavior. The proportion of T cells co-cultured with astrocytes exhibiting the poking behavior was significantly reduced (24.7% to 13.3%) upon MHC class I blocking (**Figure 6G**). When MHC class I was blocked, the percentage of scanning CD8^+^ T cells increased (61% to 68.5%) and the proportion of the dancing population slightly augmented (11% to 14.3%), but these differences did not reach statistical significance (**Figure 6G**). Our results suggest that in the absence of the TCR-MHC class I interactions, fewer CD8^+^ T cells showed the poking behavior, thus reducing synapse-like contact with inflamed astrocytes. We compared the track characteristics of three behaviors after blocking MHC class I; round cells were not analyzed given their very low number. We observed that track straightness and maximal speed achieved by the poking CD8^+^ T cells were significantly increased compared to isotype control (**SI Figure S2**). Scanning CD8^+^ T cells presented a significant increase in displacement length and a small but not significant increase in track straightness when MHC I class was blocked compared to control. Altogether, these results support the idea that the astrocytic MHC class I expression favors the synapse-like interaction with activated CD8^+^ T cells by increasing the proportion of poking CD8^+^ T cells and modeling their motility.

## Discussion

An increasing number of studies highlight species differences in both immune and neural cell responses and behaviors (14–16, 35–37). Therefore, to unravel specific mechanisms and interactions contributing to the dialogue between human lymphocytes and human glial and neuronal cells it is essential to study human cells. As a proof of concept, we have successfully established an *in vitro* co-culture model to quantify and characterize the spatiotemporal behavior of human activated CD8^+^ T cells while in contact with primary human astrocytes and neurons using time-lapse microscopy (**Figure 1**). Our multiple variable analysis showed that CD8^+^ T cells in contact with astrocytes presented a higher motility profile and an increased exploration of their environment compared to those interacting with neurons (**Figure 2**). We identified four distinct CD8^+^ T lymphocyte behaviors: scanning, dancing, poking and round in astrocyte co-cultures (**Figure 3**). Using statistical analyses of multiple spatiotemporal measures, we confirmed that each behavior exhibited a specific subset of characteristics compared to the others (**Figure 4**). Inflammation, which was mimicked by treating neural cells with IL-1β, a cytokine elevated in multiple neurological diseases (38, 39), triggered specific changes in CD8^+^ T cells. The scanning behavior was more prominent in IL-1β-treated vs. untreated astrocyte co-cultures, and specific behavior types (e.g. dancing) showed significant changes in their spatiotemporal measures (**Figure 5**). Finally, blocking the key TCR-MHC class I interaction in our co-cultures significantly diminished the proportion of CD8^+^ T cells exhibiting the poking behavior and affected specific spatiotemporal measures (e.g. speed, arrest coefficient) (**Figure 6**). Overall, our *in vitro* time-lapse model can efficiently capture changes of spatiotemporal features of human T lymphocytes interacting with human neural cells without affecting the viability of fragile cells and provides a novel and sensitive tool to investigate mechanisms involved in immune cell-tissue cell interactions in different conditions.

The complex bidirectional dialog that takes place between T lymphocytes and other cell types plays a central role in immune surveillance. Seminal work in mouse model highlighted that the contact duration between T cells and APC in lymph nodes could shape T cell activation and functions (2). Investigating this interaction with sufficient spatiotemporal resolution remains technically challenging due to very high dynamics of T cells. Many studies have used simplified *in vitro* models to characterize T cell motion. In these studies, APC were replaced by antigen-coated, antibody-coated glass coverslips, planar lipid bilayers surfaces containing ligands or MHC-peptide tetramers to activate T cells (40, 41). These models are very useful to dissect specific molecular interactions, but they do not completely recapitulate the complexity of cell to cell interfaces and lack the molecular complexity and topological deformability of dual cell surface membranes. To preserve this complexity, we took advantage of the spinning disk confocal technology to image human T cells directly interacting with human primary neural cells. Combined with non-toxic and long-lasting fluorescent dyes, fast acquisition of large z-stacks at short intervals makes it possible to visualize T cells interacting with astrocytes and neurons without employing genetic approaches while limiting phototoxicity. We tracked numerous T cells for a sufficient length of time to achieve sufficient spatiotemporal resolution, allowing us to distinguish and characterize multiple different CD8 T cell behaviors. Other microscopic techniques such as laser scanning confocal microscopy provide higher resolution but present slower temporal acquisition which do not allow capturing the diversity of immune cell motility behaviors. Notably, the traveling speed of CD8^+^ T cells we measured was in the same range (0-28 µm/min) as what has been reported by others using *in vitro* and *in vivo* (two-photo microscopy) models (5, 41, 42). By using a small (35mm) dish with four compartments, several distinct culture conditions could be monitored while using a relatively small number of precious primary neural cells and T lymphocytes. Moreover, our method was sensitive enough to acquire numerical measures of CD8^+^ T cell behavioral changes after encountering inflamed neural cells, or after specifically preventing the MHC-TCR interaction. Therefore, our assay can be used to investigate specific molecular interactions implicated in human immune surveillance while preserving the physiology of fragile human cells such as neurons.

Our statistical analyses, correlation analysis and dimension reduction of spatiotemporal data generated from each CD8^+^ T cell track (**Figures 4** **–** **6**) provided a robust demonstration that T cells can adopt diverse spatiotemporal characteristics upon their interaction with astrocytes. These analysis tools can capture the diversity of T cell spatiotemporal behaviors by combining multiple simple numerical data extracted from a commonly used imaging software, Imaris. Our analysis showed a clear separation of kinapse-like interactions (scanning cells) vs. synapse-like interactions (poking and round cells) between astrocytes and CD8^+^ T cells (**Figure 4**). Using numerical values, our approach could easily discriminate quickly moving cells from those that are engaged in stable interactions. However, such analysis did not segregate subtle behavior variations (scanning vs. dancing or poking vs. round) that we have captured by visual inspection of the time lapse. Nevertheless, the heat maps of correlation coefficients visually illustrated the different patterns of each behavior (**Figure 4B**). The addition of other spatiotemporal measures such as CD8 T cell volume or contact astrocyte-surface area over time could potentially improve the segregation of CD8 T cell subgroups based on objective numerical values. Our analysis opens the door to unbiased characterization of numerous T cell behaviors using quantitative spatiotemporal data extracted from time lapse microscopy to explore beyond human-dependent visual characterization.

Based on visual interpretation of shape and motility and supported by unbiased analyses, we identified four different CD8^+^ T cell behaviors that exhibit characteristics reminiscent of T cell kinapse or synapse interactions (4, 6, 43). Kinapse is a transient and motile interaction between a T cell and an APC while synapse exhibits long lasting contact between a T cell and an APC. Scanning CD8^+^ T cells (**Figure 3**) were predominantly associated to kinapse-like interaction with astrocytes. Scanning cells were characterized by a flattened shape and an elongated uropod and presented important directional changes. Whereas dancing CD8^+^ T cells presented an intermediate velocity, they did not presented hallmarks of kinapse interactions. Similar to scanning T cells, they also had elongated cell bodies with the presence of uropods, but did not present the same motion pattern. The “dancing” oscillation around their contact point was observed by others in T cells infiltrating organotypic slices of CNS after induction of EAE in rats (11). We believe that dancing interactions could relate to a non-classical type of kinapse interaction. Notably, the heat maps of pairwise correlations (**Figure 4B**) as well as the comparison of individual parameters (**Figure 4C**) support the notion that dancing cells exhibit properties more closely related to scanning kinapse-like cells than those with synapse-like behaviors (poking and round cells). Further investigations will be necessary to evaluate whether this behavior is specific to particular CD8^+^ T subtypes. Poking CD8^+^ T cells exhibited mostly synapse-like characteristics, with lasting interactions, a low exploratory profile and pseudopod protrusions capable of deforming astrocyte cytoplasmic membranes. Others reported that mouse activated OT-I CD8^+^ T cells showed these pseudopod protrusions upon contact with APC expressing their cognate MHC class I-peptide complex (44). Our results suggest that astrocytic MHC class I expression favors CD8^+^ T lymphocyte synapse (e.g. poking behavior) over kinapse dynamic (**Figure 6**). We can speculate that other molecules such as integrins and cell adhesion molecules are also implicated in astrocyte-T cell interactions. For example, the integrin lymphocyte function associated antigen-1 (LFA-1) on activated T cells (45) and its cognate ligand the intercellular adhesion molecule-1 (ICAM-1) can participate in kinapse-like interactions (46). Notably, elevated ICAM-1 expression by human astrocytes has been reported in neurodegenerative conditions (47–50). Our preliminary data suggest that IL-1β increases ICAM-1 on human astrocytes (data now shown). In the CNS, microglia are key producers of IL-1β in response to a large plethora of stimuli and in numerous neuropathophysiological conditions (39, 51). Moreover, CNS infiltrating activated T cells could contribute to enhance neuroinflammation by producing cytokines (e.g. interferon gamma) acting on neural cells and increasing the expression of MHC class I as well as other key molecules (52, 53).

Finally, we believe that our approach is highly versatile and could be easily adapted to a great variety of primary human cells to better characterize the complex dialogue between immune cells and organ specific cells. Our method has been used to investigate the behavior of CD8 T cells from healthy donors on human neural cells obtained from different donors. Such experimental design allows to compare T lymphocytes from different donors such as patients and healthy donors while they interact on the same neural cells and thus to dissect T cell properties. Moreover, our system could be optimized to investigate autologous interactions between immune cells and organ specific cells by using iPSC-derived neural or other cell types and immune cells isolated from the same donors. Such assays will provide meaningful knowledge on the numerous mechanisms deployed by immune cells to perform immune patrolling in different organs.

## Data Availability Statement

The raw data supporting the conclusions of this article will be made available by the authors, without undue reservation.

## Ethics Statement

The patients/participants provided their written informed consent to participate in this study.

## Author Contributions

FL and NA conceptualized the study, analyzed and interpreted data, and wrote the manuscript. FL, ACM, NF-k and YCS conducted experiments and participated to manuscript review. OT contributed to data analysis and manuscript review. AC-B contributed to imaging optimization and manuscript review. JG and EH provided access to human CNS samples. NA secured funding. All authors contributed to the article and approved the submitted version.

## Funding

This work was supported by a grant from the Multiple Sclerosis Society of Canada (MSSOC) to NA. AC obtained a joint studentship from the FRQS-MSSC. NF-k obtained a FRQS studentship. Grant number: EGID2940.

## Conflict of Interest

The authors declare that the research was conducted in the absence of any commercial or financial relationships that could be construed as a potential conflict of interest.
