# Supplementary material for: Capturing T Lymphocytes’ Dynamic Interactions With Human Neural Cells Using Time-Lapse Microscopy
Source: Front Immunol. 2021 Apr 22;12:668483. doi: 10.3389/fimmu.2021.668483 (PMC8100528; doi:10.3389/fimmu.2021.668483)
Supplement: Supplementary file 1 [file DataSheet_1.pdf]

# Supplementary Information for

## Capturing T lymphocytes dynamic interactions with human neural cells using time-lapse microscopy

Florent Lemaitre, Ana Carmena Moratalla, Negar Farzam-kia, Yves Carpentier Solorio, Olivier Tastet, Aurélie Cleret-Buhot , Jean Victor Guimond, Elie Haddad, and Nathalie Arbour

Nathalie Arbour
Email: nathalie.arbour@umontreal.ca

**This PDF file includes:**

Supplementary figures 1 to 3
Legends for Movies S1 to S10

**Other supplementary materials for this manuscript include the following:**

Movies 1 to 10

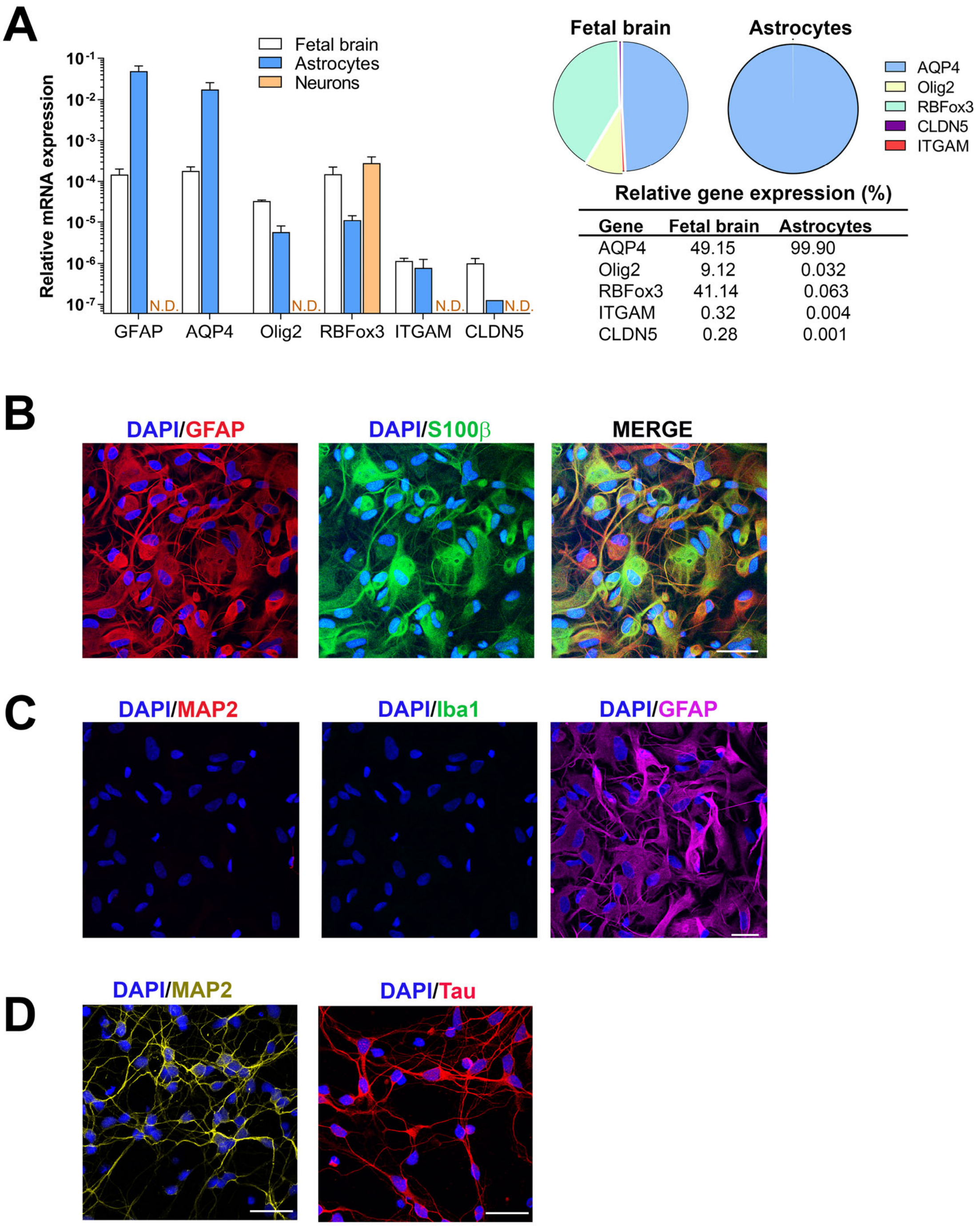


**Supplementary Figure 1: Characterization of primary cultures of human astrocytes and neurons.** A) Relative mRNA expression for each gene in fetal brain samples (n=3), isolated astrocytes (n=4), and isolated neurons (n=3). GFAP, Olig2, ITGAM and CLDN5 were below detection in RNA from isolated neurons and indicated as N.D. Relative proportions of each cell specific mRNA calculated by attributing the value of 100% to the total of mRNA relative values (AQP4+Olig2+RBFox3+ITGAM+CLDN5). Relative proportions are illustrated as pie chart and indicated in a table. B-C) Representative confocal images of immunocytochemistry for the detection of B) S100β (green) and GFAP (red) or C) MAP2 (red), Iba-1 (green), GFAP (magenta) in astrocyte cultures. D) Representative confocal images of immunocytochemistry for the detection of MAP2 and Tau in neuron culture. B-D) Nuclei were stained with DAPI (blue). Scale bare = 50µm.

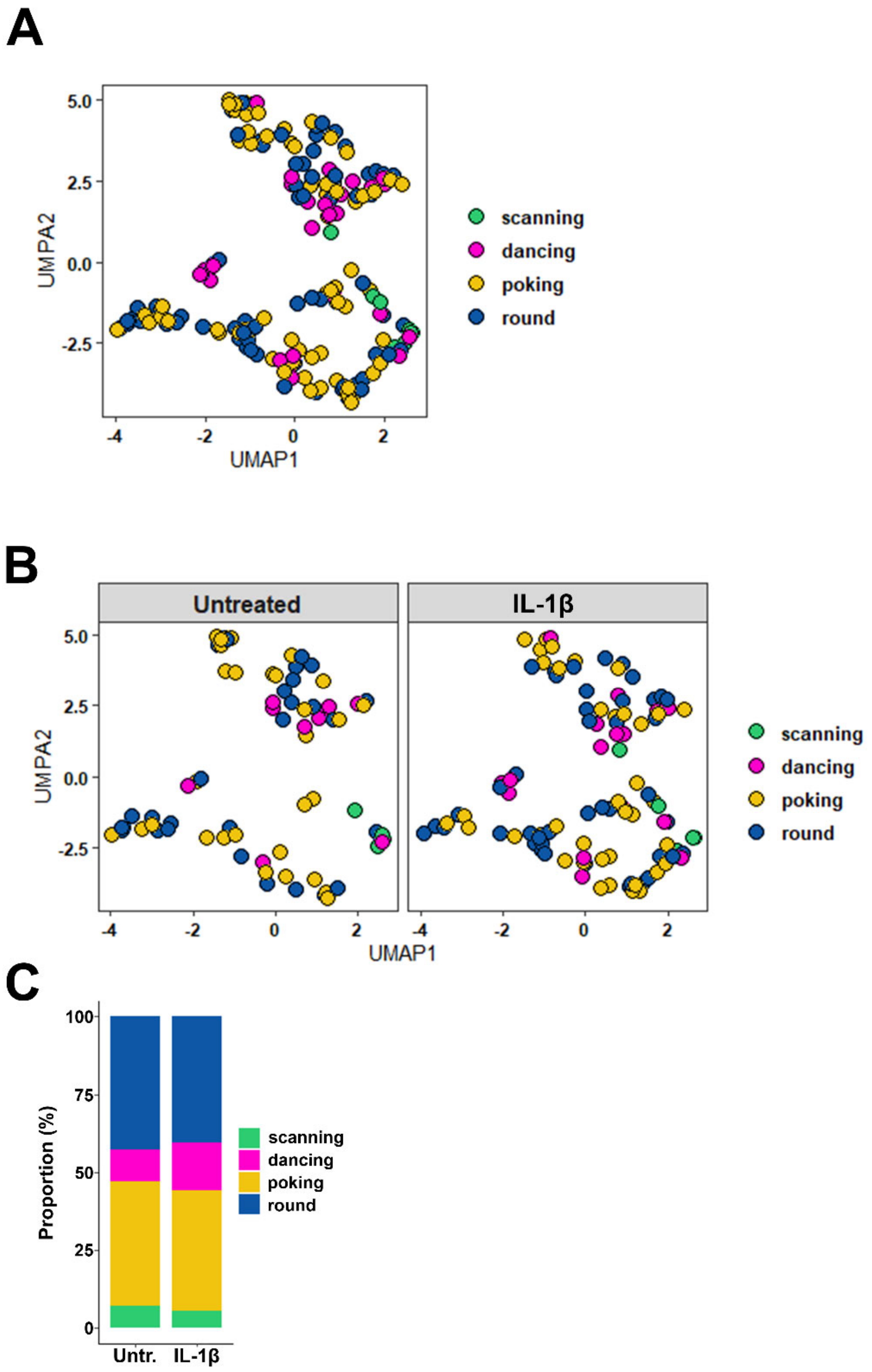


**Supplementary Figure 2**: **CD8+ T cell behaviors co-cultured on neurons.**
A-B) UMAP of CD8+ T cell behaviors co-cultured with resting and IL1β-treated neurons (A) or according to neuron treatment (B). The UMAP was performed using 10 spatiotemporal parameters measured for each CD8+ T cell. CD8+ T cell behavior was assigned to respective cell track according to visual criteria's and plotted on the UMAP. C) Percentage of CD8+ T cells exhibiting scanning, dancing, poking and round behavior in resting and IL1β-treated neuron conditions. Data are pooled from 4 distinct experiments; for each experiment a unique donor of neurons and a unique donor of CD8+ T cells were used. For each neuron-CD8+ T cell co-culture condition, 11 to 40 CD8+ T cell tracks were collected for a total of 177 tracked CD8+ T cells.

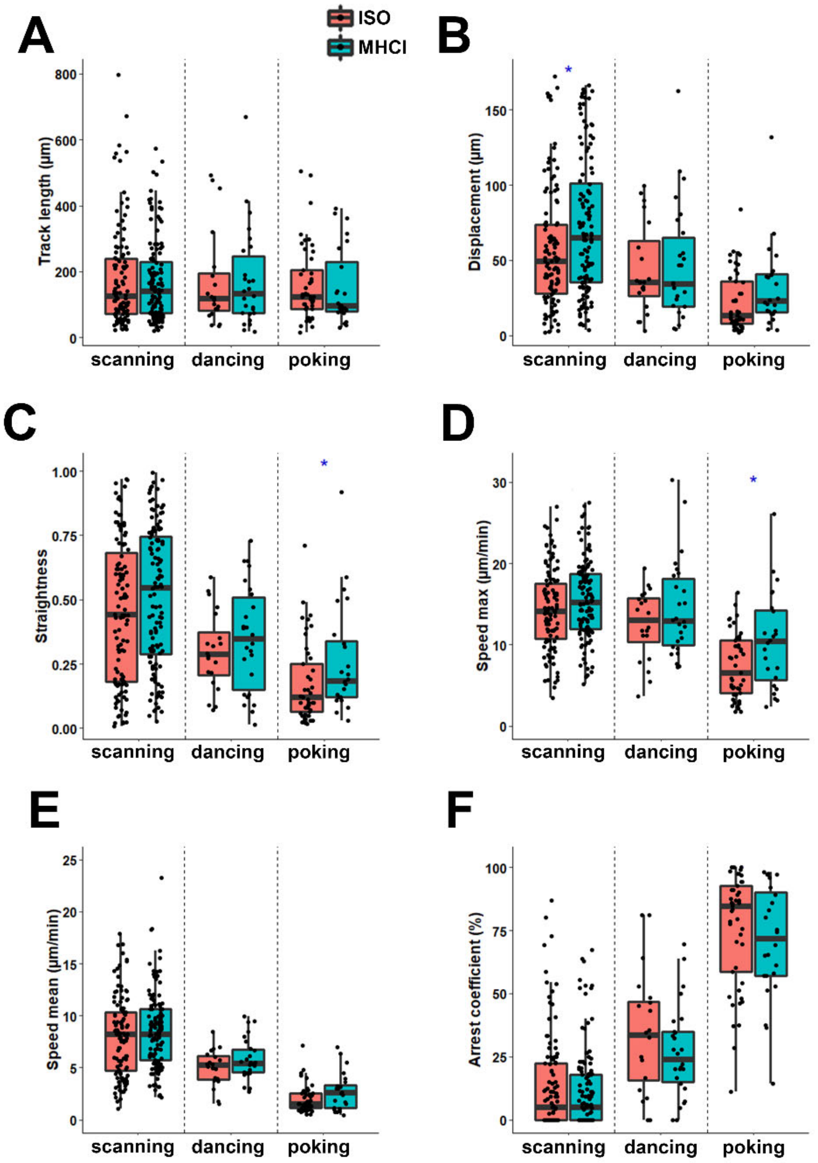


**Supplementary Figure 3**: **MHC class I expression by astrocytes impacts on specific CD8+ T lymphocyte behavior.** IL-1β-inflamed astrocytes were pre-treated with anti-MHC class I blocking antibody (MHCI) or isotype (ISO) for 30 min before starting co-culture with activated CD8+ T lymphocytes. A-F) Boxplot presenting the track length (µm), displacement (µm), straightness, maximal speed (µm/min), mean speed (µm/min) and the arrest coefficient (%) of scanning, dancing and poking CD8+ T lymphocytes. Data for astrocyte-CD8+ T cell co-cultures are pooled from 3 distinct experiments; for each experiment a unique donor of astrocytes and a unique donor of CD8+ T cells were used. For each astrocyte-CD8+ T cell co-culture condition between 39 and 76 CD8+ T cell tracks were collected for a total of 363 tracked CD8+ T cells.

**Movie 1 (separate file) :** Representative time lapse bright field microscopy of human astrocytes cultured in the microscope incubation chamber (37°C, 5% $CO_2$) over 24h. Bare scale: 20 µm, one picture per hour, 5 frame/s. (c.f Fig.1A)

**Movie 2 (separate file):** Representative time lapse bright field microscopy of human astrocytes cultured in the microscope incubation chamber (37°C, 5% $CO_2$) over 24h. Bare scale: 20 µm, one picture per hour, 5 frame/s. (c.f Fig.1B)

**Movie 3 (separate file):** Three dimensional time laps spinning disc microscopy view of activated CD8 T Lymphocytes (green) co-cultured with untreated astrocytes (magenta) over 2h. Gridlines: 20 µm, one picture per min, 20 frame/s. (c.f Fig.1D)

**Movie 4 (separate file):** Three dimensional time laps spinning disc microscopy view of activated CD8 T Lymphocytes (green) co-cultured with IL-1β-treated astrocytes (magenta) over 2h. Gridlines: 20 µm, one picture per min, 20 frame/s. (c.f Fig.1E)

**Movie 5 (separate file):** Three dimensional time laps spinning disc microscopy view of activated CD8 T Lymphocytes (green) co-cultured with untreated neurons (magenta) over 2h. Gridlines: 20 µm, one picture per min, 20 frame/s. (c.f Fig.1B)

**Movie 6 (separate file):** Three dimensional time laps spinning disc microscopy view of activated CD8 T Lymphocytes (green) co-cultured with IL-1β-treated neurons (magenta) over 2h. Gridlines: 20 µm, one picture per min, 20 frame/s.

**Movie 7 (separate file):** Three dimensional time laps spinning disc microscopy view of a reconstituted surface of scanning CD8 T cells behaviors (green) and astrocytes (magenta, transparent surface) followed over 10 min. The relative time is indicated in minutes. Gridlines: 10 µm, one picture per min, 3 frame/s. (c.f Fig. 3A)

**Movie 8 (separate file):** Three dimensional time laps spinning disc microscopy view of a reconstituted surface of dancing CD8 T cells behaviors (green) and astrocytes (magenta, transparent surface) followed over 10 min. The relative time is indicated in minutes. Gridlines: 10 µm, one picture per min, 3 frame/s. (c.f Fig. 3A)

**Movie 9 (separate file):** Three dimensional time laps spinning disc microscopy view of a reconstituted surface of poking CD8 T cell behaviors (green) and astrocytes (magenta, transparent surface) followed over 10 min. The relative time is indicated in minutes. Gridlines: 10 µm, one picture per min, 3 frame/s. (c.f Fig. 3A)

**Movie 10 (separate file):** Three dimensional time laps spinning disc microscopy view of a reconstituted surface of round CD8 T cells behaviors (green) and astrocytes (magenta, transparent surface) followed over 10 min. The relative time is indicated in minutes. Gridlines: 10 µm, one picture per min, 3 frame/s. (c.f Fig. 3A)
